# The “Needle bypass” technique: Percutaneous anatomical bypass with needle rendezvous for patients with peripheral arterial disease that have no other surgical options

**DOI:** 10.1186/s42155-021-00254-2

**Published:** 2021-08-26

**Authors:** Takuya Haraguchi, Masanaga Tsujimoto, Yoshifumi Kashima, Tsuyoshi Takeuchi, Yutaka Tadano, Daisuke Hachinohe, Umihiko Kaneko, Ken Kobayashi, Daitaro Kanno, Katsuhiko Sato, Tsutomu Fujita

**Affiliations:** 1 Department of Cardiology, Sapporo Heart Center, Sapporo, Japan; 2 Department of Cardiology and Head of Peripheral Artery Disease Center, Sapporo Heart Center, North 49, East 16, 8-1, Higashi ward, 007-0849 Sapporo, Hokkaido Japan

**Keywords:** Femoropopliteal artery disease, Bypass, Endovascular intervention, Critical limb ischemia, Peripheral arterial disease, Stent graft, Surgery

## Abstract

**Background:**

The ideal method for recanalization of complex peripheral lesions has not been determined, despite the use of the latest endovascular devices. We describe a novel method for a fully percutaneous anatomical bypass, named the “needle bypass” technique, for treatment of complex vascular lesions with failed previous surgical therapy.

**Main text:**

A 68-year-old male patient with chronic limb-threatening ischemia presented to our department. He previously had received surgical treatment 10 years prior that included the removal of the right distal common femoral artery and two surgical bypasses, an axillary-femoral bypass and an iliofemoral bypass, because he had repeated infections. He was referred to our center in order to have peripheral interventions. Since the previous conventional bridging/revascularization of the removed common femoral bifurcation had failed, the “needle bypass” technique was then used. With this novel technique, the tips of two percutaneous and bidirectional inserted needles were aligned (“needle rendezvous”) for the externalization of a guidewire in a through-and-through manner. Once this was achieved, an endovascular stent graft and an interwoven stent were deployed to cover and connect the lesion. This new technique is a minimally invasive anatomical bypass that directly connects artery to artery without any disturbance of the venous flow, and this technique, as the only option available, was performed successfully in our no-option patient.

**Conclusions:**

The “needle bypass” technique is an effective percutaneous treatment method in patients with no other surgical options.

## Background

A common finding in peripheral arterial disease is a long segment chronic total occlusion (CTO), but a recanalization with endovascular therapy (EVT) for occluded infrainguinal arteries can often be performed successfully with the latest devices and techniques. A surgical bypass (SB) for long CTOs has proven to be the most durable technique but also has significant mortality due to its complications (Zhang et al. 2014). Some cases are more difficult to treat with EVT after the failure of a surgical bypass due to the complexity of the anatomy involved. We propose a new percutaneous anatomical bypass, named the “needle bypass” technique, for high-risk patients who have no other surgical options.

## Main text

**Fig. 1:**
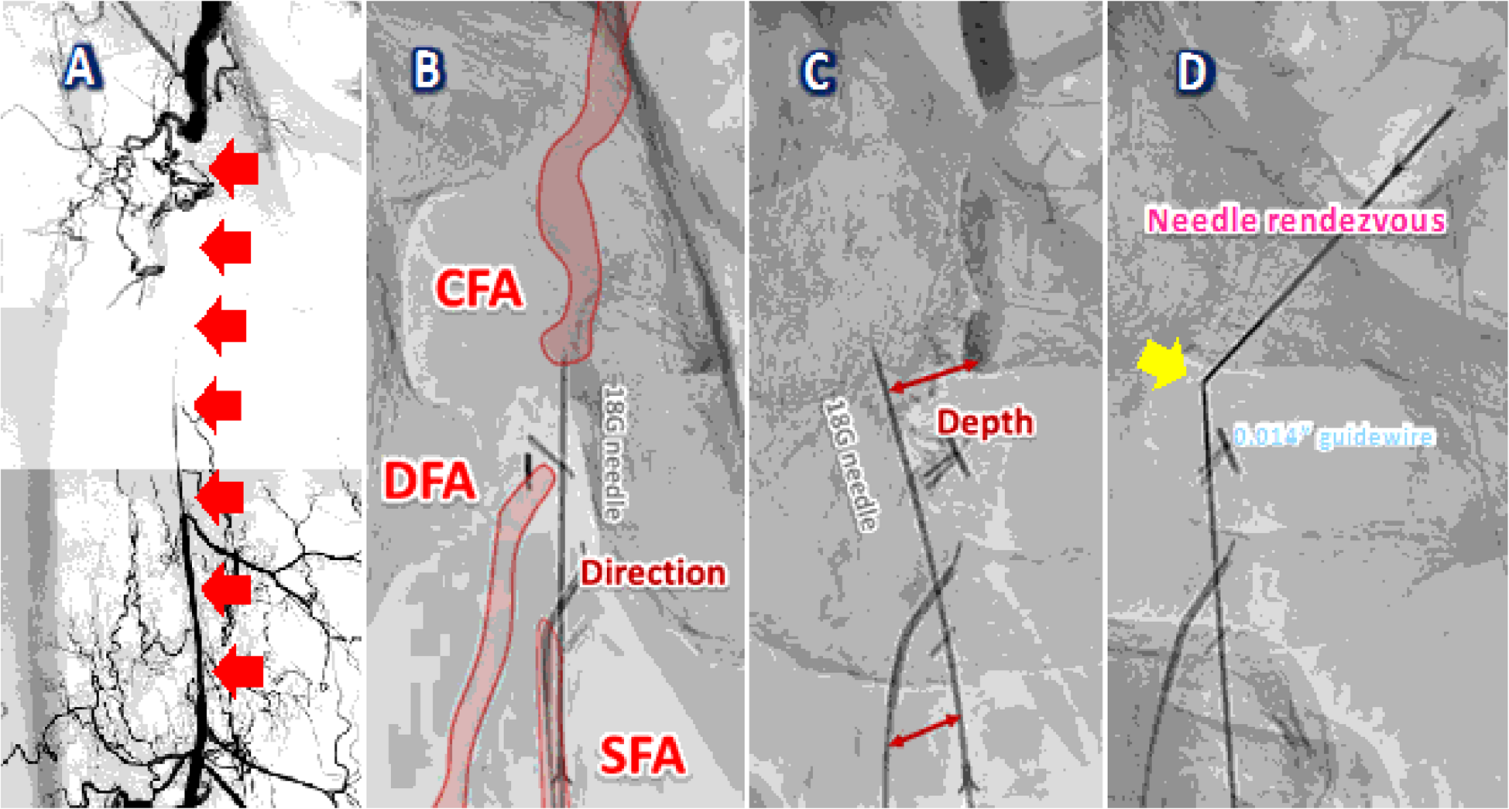
Angiography and Needle rendezvous technique. **A**. Digital subtraction angiography showed the overall lesion (red arrows). **B** and **C**. Confirming the correct direction and depth in contralateral (**B**) and ipsilateral (**C**) views. The first 18-gauge needle was used to puncture from the proximal thigh into the body below the right CFA. **D**. The second 18-gauge needle was used to puncture from the right groin through the right CFA into the first needle tip (yellow arrow). A 0.014-in. guidewire was manipulated carefully to thread two needles (“needle rendezvous”)

A 68-year-old male patient suffered from resting right foot pain due to ischemia and was referred to our department. He had a history of right common femoral artery (CFA), an axillary-femoral bypass, and an iliofemoral bypass transection due to repeated methicillin-resistant Staphylococcus aureus infections after a Miles’ operation and a lymphadenectomy for anal cancer 10 years prior. The ankle brachial index was 0.59 in the right leg. The patient was admitted to undergo EVT due to complications from the previous surgical procedures. A computed tomography angiogram (CTA) showed that there were some skin defects above the previous removal due to the bypasses, and that there were no arteries present from the distal CFA to the proximal superficial femoral artery (SFA) in his right leg due to the previous surgical ligation. A contrast effect was found from the stenotic middle SFA to the normal distal vessel. The lesion was anatomically complex and difficult to treat, but the bidirectional approach was performed in this patient. A 7-Fr guiding sheath (Destination®, Terumo Co., Japan) was used as antegrade crossover approach and a 6-Fr guiding sheath (Parent Cross®, Medikit Co. Ltd., Japan) was used as a retrograde approach. These were inserted via the left CFA and right popliteal artery, respectively. The angiographical imaging showed an overview of the lesion as a CTA (Fig. 1 A). We were not able to advance several hard guidewires including the tail of the 0.035-in. wire supported with a 4-Fr catheter (Tempo®, Cardinal Health Inc., USA) into the lesion and into the tissue near the vessel due to the scar tissue formed from the repeated infections and the previous surgical procedures. Since these lesions were too difficult to treat with a conventional intervention, the “needle bypass” technique was attempted as a novel percutaneous anatomical bypass.

**Fig. 2:**
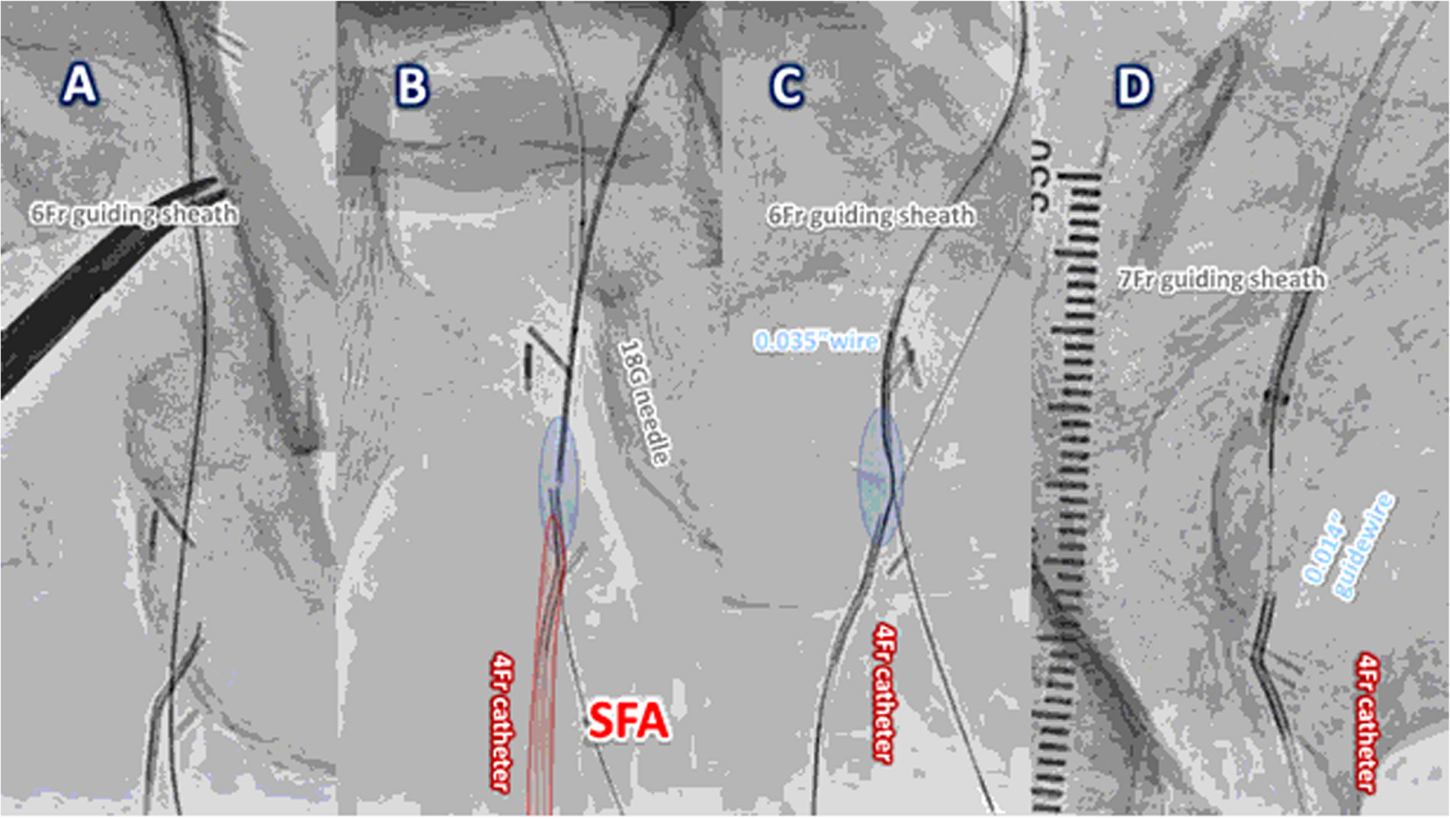
The treatment process of Needle Bypass technique. **A**. An antegrade 6-Fr guiding sheath was inserted over the pull-through guidewire into the body between the puncture sites. **B**. The third 18-gauge needle punctured from the proximal site through the space (blue frame), which was formed by a 4.0 × 20-mm semicompliant balloon dilatation, into the SFA lumen where a retrograde 4-Fr catheter was inserted. **C**. After the guidewire externalization with 0.014-in. guidewire between the third needle and 4-Fr catheter, the 4-Fr catheter was advanced into the space (blue frame) on a 0.035-in. guidewire and together advanced into the antegrade 6-Fr guiding sheath. **D**. After removal of the 6-Fr guiding sheath, a 0.014-in. guidewire inside the 4-Fr catheter was advanced into an antegrade 7-Fr guiding sheath from the left CFA as a contralateral approach

After the administration of local anesthesia at the sites of the punctures, the first 18-gauge needle (Terumo Co., Japan) was used to puncture the tissue starting at the proximal thigh into the body to below the proximal true lumen of the CFA (Fig. 1B, and C), and the second 18-gauge needle was used to puncture the tissue from the right groin through right CFA to the proximal CTO end. We confirmed the position of the needle tip inside the CFA due to the return of blood, and we continued to insert the second needle until it met the tip of the first needle. Then, a 0.014-in. guidewire was carefully used to thread the two needles to achieve a guidewire externalization that extended from needle to needle, which we named the “needle rendezvous” technique (Fig. 1D). An antegrade 6-Fr Parent Cross® sheath was inserted over the pull-through guidewire between the puncture sites (Fig. 2 A). A 4.0 × 20-mm semicompliant balloon (Sterling®, Boston Scientific Co., USA) was used to dilate the tissue in front of the 6-Fr guiding sheath to create a space, and a third 18-gauge needle was used to puncture the tissue from the proximal site through the mentioned space into the SFA lumen where the retrograde 4-Fr catheter was positioned (Fig. 2B). Furthermore, a 0.014-in. guidewire was advanced into the 4-Fr catheter through the third needle to perform a guidewire externalization.

A 0.035-in. guidewire placed inside the 4-Fr catheter was manipulated into the antegrade 6-Fr guiding sheath (Fig. 2 C), which was consequently retracted into the CFA. After the removal of the 6-Fr guiding sheath, the 4-Fr catheter was advanced into an antegrade 7-Fr guiding sheath from the left CFA as a contralateral approach to accomplish a true guidewire externalization (Fig. 2D).

To treat complex lesions (including the extravascular route), the “Pave-and-Crack” technique was intentionally performed, and this technique facilitates the safe introduction and effective scaffolding of stent grafts through a lesion access followed by aggressive balloon dilatation (Hinchliffe et al. 2007; Dias-Neto et al. 2018), The lesion was aggressively dilated with a 7.0 × 40-mm noncompliant balloon (SHIDEN HP®, Kaneka Co., Japan) and fully covered with a 7.0 × 250-mm stent graft (Viabahn®, W.L. Gore & Associated, Inc., USA). In this case, it was inevitable to implant an interwoven stent (Supera®, Abbott Vascular, USA), which provides a higher radial force to resist any recoil or extrinsic compression from the solid tissue in the extravascular site and from any hip joint motion.

**Fig. 3:**
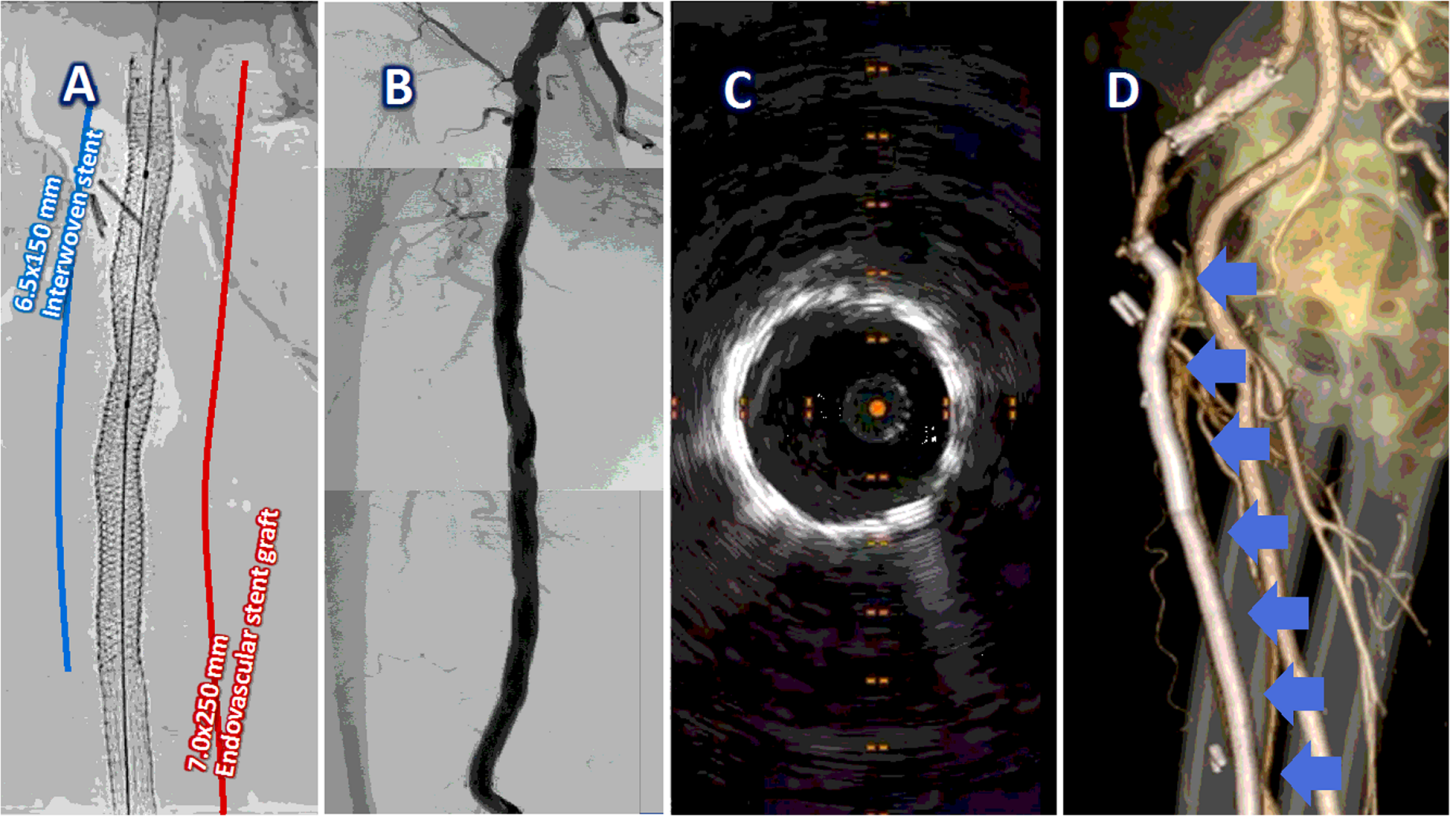
Final results and computed tomography angiogram at the 12-month follow-up. **A**. The lesion was fully covered with a 7.0 × 250-mm endovascular stent graft (red line), and a 6.5 × 150-mm interwoven stent (blue line) was implanted to resist recoil and extrinsic compression. **B**. Final angiogram demonstrates a restored blood flow. **C**. Intravascular ultrasound showed symmetrically expanded scaffolds. **D**. Computed tomography angiogram found patency (blue arrows) one year after the treatment

After the 6.5 × 150-mm interwoven stent implantation and the postballoon dilation with a 7.0-mm noncompliant balloon by using the highest pressure, an angiogram and an intravascular ultrasound finally demonstrated the success of our “needle bypass” technique to perform percutaneous anatomical bypass, and we did not have any complications from the technique (Fig. 3 A-C). The patient’s symptoms and physiological examinations were largely improved after the procedure, and there was no patency loss, no further need for reintervention, and no stent thrombosis in this patient one year after the technique (Fig. 3D).

## Discussion

We describe the “needle bypass” technique, which is a percutaneous anatomical bypass using a “needle rendezvous” technique, and this technique is a novel recanalization procedure that was first described in this case report. This crossing technique can revascularize the artery previously removed from surgery or trauma. A two-stage puncture technique to shorten the lesion length was required for this technique to be successful in this patient because the needle-directed puncture from the CFA to the middle SFA was difficult due to the shallow depth between both arteries. The type of scaffold needed in this technique depends on the lesion stiffness and the anatomy. We implanted a combination of endovascular stent grafts and interwoven stents for this patient due to the complex anatomy from the previously failed SFA. Stent grafts alone may be all that is needed in less complicated lesions when using this technique.

Determining the success of any treatment for a failed SB is often difficult due to the variety of the treatment options available (Hagino et al. 2007), but there are some other reconstruction techniques for patients who have no other options. One of the newer techniques is the PQ DETOUR system (PQ Bypass®, Inc., Sunnyvale, CA, USA), and this technique is also a fully percutaneous bypass technique with a TORUS stent graft implantation (Krievins et al. 2018). This unique crossing device is used to cross from the proximal SFA into the deep femoral vein and back into the popliteal artery. The DETOUR1 trial evaluated the outcomes, including the 1-year safety and effectiveness, that were associated with this system. This trial demonstrated that the rates of the 1-year primary patency, the secondary patency, and freedom from a stent graft thrombosis were 81 %±4 %, 90 %±3 %, and 84 %±4 %, respectively, but deep venous thromboses (DVT) developed in 2 of 79 target limbs (3 %) within 1 year (Krievins et al. 2020). In Japan, this system has not been approved for use in daily practice, therefore, we invented a new treatment method and technique for complex lesions. Nakama et al. described the percutaneous endoluminal bypass procedure via the veins for an iliac artery occlusion in cases that were not candidates for conventional EVT (Nakama et al. 2020). These procedures included the arterial reconstruction via veins, but this technique may lead to the development of DVTs. The needle bypass technique connects artery to artery directly without the venous flow disturbance which often leading to DVTs.

The main limitation of this technique is that it must be performed below the inguinal ligament, not within the intraperitoneal cavity, which leads to the complications. Also, dedicated devices to standardize this technique need to be produced. Recently, this technique was successfully used in three cases without complications.

## Conclusions

The “needle bypass” technique, which is the use of percutaneous anatomical bypass with rendezvous needles, is a safe and effective novel percutaneous treatment option in patients with no other surgical options. The long-term outcomes after using this technique have not yet been fully studied, and careful patient follow-up is mandatory following this procedure.

## Data Availability

The datasets used and/or analyzed during the current study are available from the corresponding author on reasonable request.

## Abbreviations

CTO: Chronic total occlusion
EVT: Endovascular therapy
SB: Surgical bypass
CFA: Common femoral artery
CTA: Computed tomography angiogram
SFA: Superficial femoral artery
DVT: Deep venous thrombosis

## References

[CR1] Dias-Neto M, Matschuck M, Bausback Y, Banning-Eichenseher U, Steiner S, Branzan D, Staab H, Varcoe RL, Scheinert D, Schmidt A (2018). Endovascular treatment of severely calcified femoropopliteal lesions using the “pave-and-crack” technique: technical description and 12-month results. J Endovasc Ther.

[CR2] Hagino RT, Sheehan MK, Jung I, Canby ED, Suri R, Toursarkissian B (2007). Target lesion characteristics in failing vein grafts predict the success of endovascular and open revision. J Vasc Surg.

[CR3] Hinchliffe RJ, Ivancev K, Sinesson B (2007). ‘‘Paving and Cracking’’: An Endovascular Technique to Facilitate the Introduction of Aortic Stent-Grafts Through Stenosed Iliac Arteries. J Endovasc Ther.

[CR4] Krievins D, Savlovskis J, Ezite N, Hill A, Kisis K, Gedins M, Zellans E, Holden A (2018). The DETOUR procedure: no more need for conventional bypass surgery?. J Cardiovasc Surg (Torino).

[CR5] Krievins DK, Halena G, Scheinert D, Savlovskis J, Szopiński P, Krämer A, Ouriel K, Nair K, Holden A, Schmidt A (2020). One-year results from the DETOUR I trial of the PQ Bypass DETOUR System for percutaneous femoropopliteal bypass. J Vasc Surg.

[CR6] Nakama T, Obunai K, Muraishi M (2020). Percutaneous endoluminal anatomical bypass for patients with external iliac artery occlusion after failed conventional endovascular recanalization. Catheter Cardiovasc Interv.

[CR7] Zhang JQ, Curran T, McCallum JC, Wang L, Wyers MC, Hamdan AD, Guzman RJ, Schermerhorn ML (2014). Risk factors for readmission after lower extremity bypass in the American College of surgeons national surgery quality improvement program. J Vasc Surg.

